# Cyclic diguanylate riboswitches control bacterial pathogenesis mechanisms

**DOI:** 10.1371/journal.ppat.1007529

**Published:** 2019-02-07

**Authors:** Rita Tamayo

**Affiliations:** Department of Microbiology and Immunology, University of North Carolina at Chapel Hill School of Medicine, Chapel Hill, North Carolina, United States of America; Nanyang Technological University, SINGAPORE

Many bacterial species use cyclic diguanylate (c-di-GMP) to control their physiology and behaviors in response to extracellular stimuli. C-di-GMP primarily mediates the switch between a free-living, motile state and a nonmotile lifestyle, with the latter often entailing surface adherence and biofilm development [1, 2]. This paradigm is conserved across diverse gram-positive and gram-negative species, though the specific extracellular signals that impact c-di-GMP levels and the response mechanisms employed vary. Because c-di-GMP affects motility and adherence, it consequently can influence bacterial pathogenicity [3]. In addition, a number of pathogenic bacteria have engaged c-di-GMP signaling to control specific virulence mechanisms, including effector secretion mechanisms and toxin biosynthesis [4–7].

## How are intracellular c-di-GMP levels controlled?

The extracellular signals that influence bacterial c-di-GMP metabolism are largely undefined. *Salmonella enterica* serovar Typhimurium alters its c-di-GMP content in response to glucose, N-acetylglucosamine, sialic acid, or arginine [8]. Genetic evidence indicates that *Vibrio cholerae* reduces its intracellular c-di-GMP levels upon infection of the small intestine [4, 9], and bile and bicarbonate provide physiologically relevant host cues to regulate c-di-GMP levels [10]. In *Clostridioides difficile* (also *Clostridium difficile*), at least one c-di-GMP hydrolytic enzyme, PdcA, is induced in response to nutrient limitation through the nutrient-responsive transcriptional regulator CodY present in many gram-positive species [11].

In response to extracellular stimuli, bacteria alter intracellular c-di-GMP concentrations through the opposing activities of diguanylate cyclases (DGCs) that synthesize c-di-GMP from guanosine triphosphate (GTP), and phosphodiesterases (PDEs) that hydrolyze c-di-GMP. Many bacteria, particularly the Gammaproteobacteria, encode numerous DGCs and PDEs [1]. Changes in c-di-GMP thus are likely accomplished by adjusting the production, activity, or localization of these enzymes so that only a subset of the enzymes function under a particular environmental condition. *C*. *difficile* strain 630 encodes 18 confirmed or putative DGCs and 17 confirmed or putative PDEs [12, 13]. Most of the enzymes are predicted to be membrane-localized, and several contain additional domains that may modulate enzymatic activity [12]. If and how these features impact c-di-GMP synthesis and hydrolysis is difficult to predict and must be determined experimentally. PdcA, for example, contains a PAS domain that is required for c-di-GMP hydrolysis and is postulated to be involved in nutrient sensing [11].

## How are changes in intracellular c-di-GMP levels sensed?

C-di-GMP levels are sensed by specific intracellular receptors. Several protein sensors of c-di-GMP have been identified (reviewed in [14]), and the regulatory consequence of c-di-GMP binding is determined by features of the specific receptor protein. For instance, c-di-GMP–responsive transcription factors show altered DNA binding capacity in response to the ligand, resulting in changes in gene expression. Other c-di-GMP receptors act posttranslationally to modulate the activity of other proteins or protein complexes, such as the PilZ domain proteins that inhibit swimming motility by interfering with flagellar motor function [15, 16].

**Fig 1:**
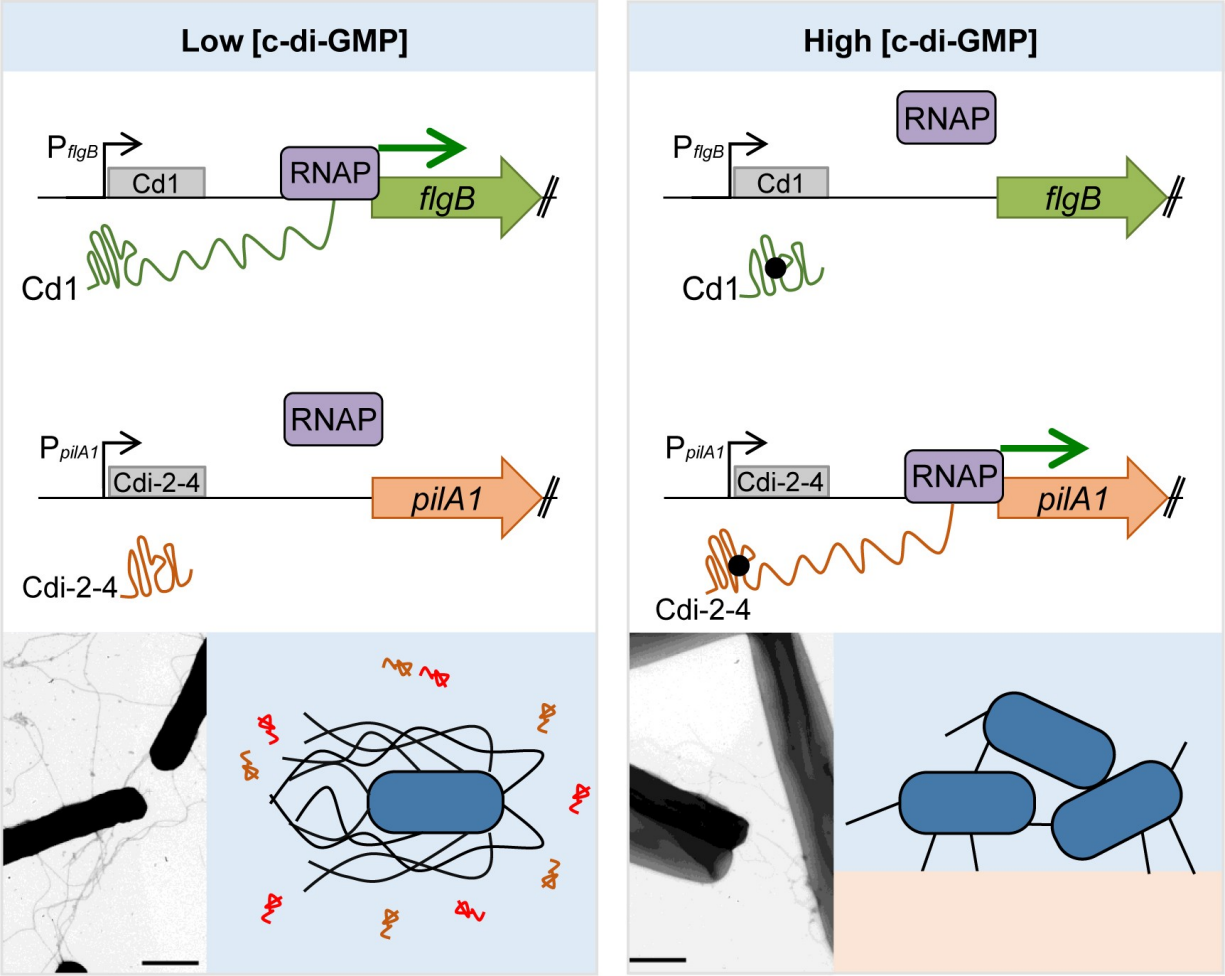
Model for c-di-GMP mediated transition between free-living, toxin producing state and an adherent state in *Clostridium difficile*. (Left) Under low intracellular c-di-GMP conditions, the Cd1 “off” riboswitch forms a structure conducive to transcription of the downstream *flgB* operon. The Cdi-2-4 “off” riboswitch assumes a structure that includes a transcription terminator, precluding transcription of downstream TFP genes. Therefore, low c-di-GMP favors flagellated, swimming bacteria that also produce the glucosylating toxins TcdA and TcdB. (Right) Elevated intracellular c-di-GMP (represented by black dots) results in ligand binding to the Cd1 and Cdi-2-4 riboswitches, causing mRNA conformations that terminate transcription of the *flgB* mRNA but promote transcription read-through of *pilA1* and downstream TFP genes. High c-di-GMP thus results in nonflagellated, nontoxigenic, piliated bacteria with increased adherence properties. Presumably intermediate c-di-GMP concentrations exist that result in bacteria with both flagella and TFP. (Insets) Transmission electron micrographs and graphical depictions of *C*. *difficile* 630Δerm with basal c-di-GMP resulting in flagellated, toxin-secreting bacteria (left) or elevated c-di-GMP resulting in nontoxigenic bacteria bearing Type IV pili (right). Toxins TcdA and TcdB are represented by red and orange symbols. Cd1, *flgB* riboswitch; Cdi-2-4, *pilA1* riboswitch; c-di-GMP, cyclic diguanylate; mRNA, messenger RNA; TFP, type IV pili.

Many bacteria encode RNA-based c-di-GMP sensors. Riboswitches are encoded in the 5′ leader sequence (untranslated region [UTR]) of some messenger RNAs (mRNA), and they fold to adopt a structure that binds a specific ligand [17]. Ligand binding by the nascent mRNA causes the formation of an alternate, mutually exclusive structure and typically affects whether the downstream genes are expressed or translated. For example, ligand binding can affect the formation of an intrinsic transcription terminator within the 5′ UTR, determining whether the downstream gene is transcribed (Fig 1). Alternatively, the RNA structure assumed can affect the accessibility of the ribosome binding site, influencing translation of the downstream coding sequence. Depending on the specific riboswitch and its genetic context, riboswitches can act as “on” or “off” switches in response to ligand binding, promoting or preventing gene expression, respectively. Two classes of c-di-GMP specific riboswitches have been identified: class I and class II [18, 19]. Typically, riboswitches bind ligands that are the products of the regulated pathway, serving as feedback mechanisms for that metabolite. The c-di-GMP riboswitches are unusual in that they rarely control c-di-GMP metabolic genes. Instead, c-di-GMP riboswitches are encoded upstream of genes known or predicted to be involved in motility, chemotaxis, adherence, or other processes that are often targets of c-di-GMP regulation. In fact, the class I riboswitches are also termed genes for the environment, membranes and motility (GEMM) riboswitches, for reflecting this conserved function of c-di-GMP riboswitches [20].

## How do class I and class II riboswitches respond to c-di-GMP?

Only a small subset of c-di-GMP riboswitches have been validated [18, 19, 21–26], and even fewer have been studied in their native genetic context. The best studied system occurs in the gram-positive bacterium *Clostridium difficile*, a major cause of antibiotic-associated, cytotoxin-mediated diarrhea in humans. *C*. *difficile* possesses a particularly large number of c-di-GMP riboswitches. The *C*. *difficile* 630 genome contains 12 class I and 4 class II c-di-GMP riboswitches [18, 19]. Only 11 of the riboswitches are functional, i.e., the expression of the downstream genes is regulated by c-di-GMP [25, 26]. These functional riboswitches are highly conserved across *C*. *difficile* strains, unless the regulated genes are also absent; the nonfunctional riboswitches are less well-conserved [26]. In *C*. *difficile*, the seven functional class I riboswitches are uniformly “off” switches, with elevated c-di-GMP triggering transcription termination and precluding expression of the downstream genes. Conversely, the four class II riboswitches are “on” switches, with elevated c-di-GMP favoring gene expression. However, in the soil-dwelling, gram-positive bacterium *Bacillus thuringiensis*, the class I riboswitch Bc2 acts as an “on” switch by promoting transcription read-through of the *cap* gene encoding collagen adhesion protein [27]. The gram-negative *Vibrio cholerae* encodes two class I c-di-GMP riboswitches that respond to the ligand in opposing directions, and neither function by controlling transcription termination [21, 22]. Therefore, regulatory attributes cannot necessarily be extrapolated to c-di-GMP riboswitches in other bacterial species but must be evaluated in each context.

## How does c-di-GMP signaling through riboswitches impact pathogenesis?

Because its genome contains a large number of c-di-GMP riboswitches regulating genes with measurable phenotypes, *C*. *difficile* serves as an excellent model for understanding how c-di-GMP riboswitches function in vivo. *C*. *difficile* produces peritrichous flagella that are involved in motility and intestinal colonization [28]. As in other species, flagellar gene expression is coordinated in a hierarchical manner, and “early stage” flagellar genes are coexpressed as part of the 23 kb *flgB* operon. A class I riboswitch, Cd1 (also named Cdi-1-3), lies in the 5′ UTR of the *flgB* operon (Fig 1) [18]. Initial studies using reporter fusions to the riboswitch region, assayed in *Escherichia coli*, indicated that Cd1 functions as an “off” switch in response to c-di-GMP [18]. Consistent with this, in vitro transcription assays showed that c-di-GMP increased the appearance of a terminated product in a dose-dependent manner [18]. Subsequent work in *C*. *difficile* demonstrated that increasing intracellular c-di-GMP inhibits *flgB* operon expression [29], and a truncated RNA corresponding to the riboswitch-mediated termination product was detected by northern blot [25, 30]. Consistent with “off” function, c-di-GMP inhibits flagellum biosynthesis and swimming motility of *C*. *difficile* [29]. Expression of the genes encoding the glucosylating toxins that are essential for disease development, *tcdA* and *tcdB*, is linked to flagellar gene expression [31]. The sigma factor SigD not only coordinates flagellar gene transcription but also activates transcription of *tcdR*, which encodes another sigma factor required for *tcdA* and *tcdB* expression. The *sigD* gene is encoded within the *flgB* operon, and its expression is therefore controlled by c-di-GMP via the Cd1 riboswitch [29]. Accordingly, c-di-GMP indirectly inhibits expression of *tcdR*, *tcdA*, and *tcdB*, resulting in reduced toxin production and cytotoxicity [5]. These findings suggest that c-di-GMP must be sufficiently low to allow motility and toxin production by *C*. *difficile* during infection.

In contrast, c-di-GMP production must be upregulated to stimulate adherent behaviors. *C*. *difficile* produces type IV pili (TFP) that contribute to autoaggregation, biofilm formation, adherence to epithelial cells, and persistence in a mouse model of infection [23, 32, 33]. C-di-GMP promotes the expression of TFP genes via a class II riboswitch upstream of *pilA1*, which encodes the major pilin subunit of TFP (Fig 1) [23]. This riboswitch, Cdi-2-4, assumes a structure in the presence of c-di-GMP that allows transcription of the *pilA1* coding sequence, as well as some read-through of the downstream *pilB* operon encoding other TFP components. In the absence of c-di-GMP, Cdi-2-4 folds to induce transcription termination, thus preventing TFP gene expression, biosynthesis, and adherence.

Additionally, the expression of CD630_28310 and CD630_32460 is also positively regulated by c-di-GMP via class II riboswitches, Cdi-2-3 and Cdi-2-1, respectively [19, 24]. These genes encode sortase-dependent surface proteins that are predicted to act as adhesins [19, 24, 34]. Notably, the zinc-dependent metalloprotease ZmpI (also named Pro-Pro endopeptidase PPEP-1) cleaves the CD630_28310 and CD630_32460 proteins near their cell wall anchor motifs, releasing them from the bacterial surface [35]. A *C*. *difficile zmpI* mutant is attenuated in a hamster model of infection [34], presumably because it is defective for release of CD630_28310 and/or CD630_32460 from the cell surface. The *zmpI* mRNA (CD630_28300) contains a class I riboswitch and is negatively regulated by c-di-GMP. The opposing regulation of ZmpI and its surface protein targets has been proposed to integrate increased c-di-GMP to simultaneously promote production of the surface proteins and inhibit their release by ZmpI [36]. Together these studies indicate that, as in other bacterial species, *C*. *difficile* uses c-di-GMP to regulate the transition between motile and nonmotile lifestyles and to control virulence factors.

## What can we learn about c-di-GMP signaling by studying c-di-GMP riboswitches?

*C*. *difficile* appears to rely primarily on c-di-GMP riboswitches rather than protein sensors, presenting opportunities to address outstanding questions in c-di-GMP signaling. For example, given multiple physiological targets in a single bacterial species, how is c-di-GMP regulation coordinated? C-di-GMP riboswitches in *C*. *difficile* appear to display a range of response kinetics to c-di-GMP, suggesting hierarchical activation or inhibition of gene expression from given changes in c-di-GMP [26]. The set of riboswitches found in a single species provides a means to test as proof-of-principle that different intracellular concentrations of c-di-GMP trigger specific responses. A hierarchical response to c-di-GMP is supported by a recent study measuring the transcription of riboswitch-regulated genes to increasing intracellular c-di-GMP [26]. Additionally, given the pleiotropic role of c-di-GMP, how is its control of individual phenomena achieved? Riboswitch-mediated regulation occurs in cis, so defined mutations in riboswitches that prevent c-di-GMP binding would allow targeted abrogation of regulated outcomes. Riboswitches are also being implemented as biosensors in a number of systems to measure native c-di-GMP levels under different growth conditions [32, 37, 38]. This approach offers greater sensitivity than quantification of c-di-GMP in cellular extracts, as well as the ability to examine c-di-GMP on a single-cell basis rather than in the bulk population. The availability of “on” and “off” riboswitches with characterized (and alterable) affinities for c-di-GMP may provide modular systems that can be utilized as biosensors in a wide range of bacterial species.

Finally, a number of other pathogenic bacteria encode putative c-di-GMP riboswitches [18, 19]. *Vibrio parahaemolyticus* and *V*. *vulnificus* encode homologues of Vc1 and Vc2 from *V*. *cholerae* upstream of the *gbpA* and *tfoX* orthologues, respectively. *Bacillus anthracis* encodes a c-di-GMP riboswitch upstream of a putative methyl accepting chemotaxis protein, suggesting a role in chemotaxis and motility. The opportunistic pathogen *Aeromonas hydrophila* encodes c-di-GMP riboswitches upstream of putative fimbrial and chitinase genes, implying roles in adherence. The *Clostridium tetani* and *C*. *perfringens* genomes contain multiple c-di-GMP riboswitches, including upstream of orthologous genes encoding a predicted collagen binding domain. Additional work is needed to elucidate the role of c-di-GMP riboswitches in modulating the physiology and behavior of bacterial pathogens.
